# Governing HPV-related carcinoma using vaccines: Bottlenecks and breakthroughs

**DOI:** 10.3389/fonc.2022.977933

**Published:** 2022-09-13

**Authors:** Rahul Bhattacharjee, Lamha Kumar, Archna Dhasmana, Tamoghni Mitra, Abhijit Dey, Sumira Malik, Bonglee Kim, Rohit Gundamaraju

**Affiliations:** ^1^ Department of Cell and Developmental Biology, Sackler School of Medicine, Tel Aviv University, Tel Aviv, Israel; ^2^ School of Biology, Indian Institute of Science Education and Research, Thiruvananthapuram, India; ^3^ Himalayan School of Biosciences, Swami Rama Himalayan University, Dehradun, India; ^4^ KIIT School of Biotechnology, Kalinga Institute of Industrial Technology (KIIT-DU), Bhubaneswar, Odisha, India; ^5^ Department of Life Sciences, Presidency University, Kolkata, West Bengal, India; ^6^ Amity Institute of Biotechnology, Amity University Jharkhand, Ranchi, Jharkhand, India; ^7^ Department of Pathology, College of Korean Medicine, Kyung Hee University, Seoul, South Korea; ^8^ ER Stress and Mucosal Immunology Lab, School of Health Sciences, University of Tasmania, Launceston, TAS, Australia

**Keywords:** vaccines, human papillomavirus, cervical cancer, viral cancers, therapeutic vaccines

## Abstract

Human papillomavirus (HPV) contributes to sexually transmitted infection, which is primarily associated with pre-cancerous and cancerous lesions in both men and women and is among the neglected cancerous infections in the world. At global level, two-, four-, and nine-valent pure L1 protein encompassed vaccines in targeting high-risk HPV strains using recombinant DNA technology are available. Therapeutic vaccines are produced by early and late oncoproteins that impart superior cell immunity to preventive vaccines that are under investigation. In the current review, we have not only discussed the clinical significance and importance of both preventive and therapeutic vaccines but also highlighted their dosage and mode of administration. This review is novel in its way and will pave the way for researchers to address the challenges posed by HPV-based vaccines at the present time.

## Introduction

**Figure 1:**
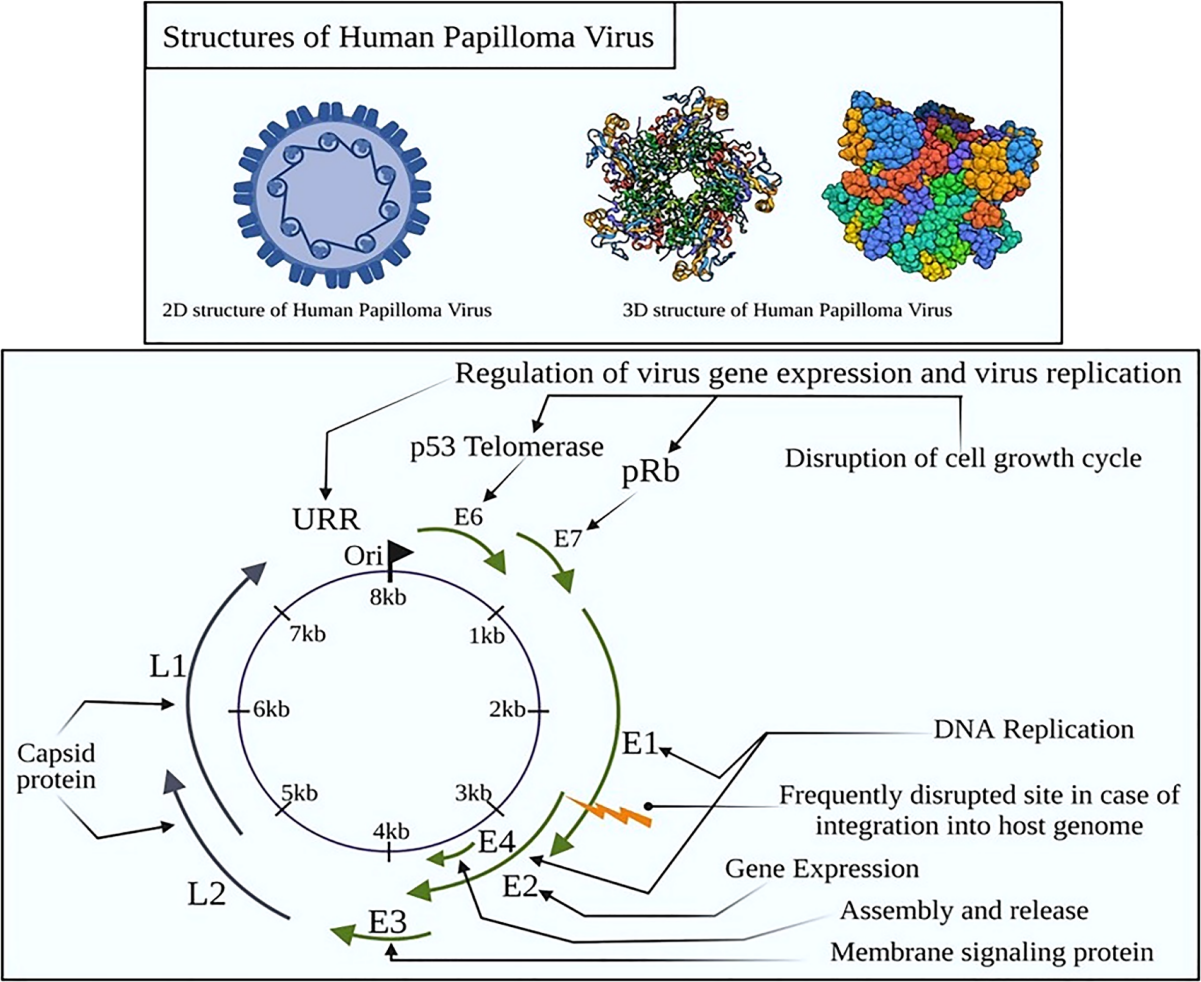
Structural organization of HPV with early and late proteins. E proteins are non-structural and have a role in viral genome duplication and expression although the L proteins form the capsid of the intact virion. In cervical cancer, these proteins activate the oncogenes to activate telomerase, inducing abnormal centrosome duplication by the inactivation of p53 and retinoblastoma (Rb) tumor suppressor genes.

At the global platform, human papillomavirus (HPV) is reported as one of the the most prevalent sexually transmitted infection (STI), having a significant detrimental impact on the social life of an individual (1). Most of the sexually active men and women will be infected through recurrent infections depending upon the immunity of an individual (2). HPV is a non-enveloped, small, double-stranded DNA virus that belongs to the family *Papillomaviridae* (3). The circular HPV genome constitutes 7,500–8,000 bp of DNA linked to histones and is compacted into chromatin-like aggregates (**Figure 1**).

HPVs infects basal cells of epithelial tissue and causes either malignant or benign lesions of the mucosal layer of the gastrointestinal tract, upper aerodigestive tract and the anogenital tract, and skin (4). Cervical and anogenital cancers are linked to high-risk HPV infections and are the most common HPV-related diseases. HPV infection has a significant occurrence in the majority of cervical cancers (5). According to the GLOBOCAN reports, the third most predominant cancer in women is cervical cancer (6). There were an estimated 604,127 cases of cervical cancer reported worldwide in 2020, which accounts for 3.1% of the global cancer cases. A total of 341,831 deaths were reported because of cervical cancer, which accounts for 3.3% of the global caseload (7). According to the WHO, HPV causes more than 95% of cervical cancer primarily as an outcome of dysbiosis of early and late proteins as most widespread viral infection of the female reproductive system (8). HPV viruses can be divided into high-risk HPVs and the low-risk HPVs. High-risk HPVs (HR-HPVs) cause oropharyngeal (throat, tonsil, and oral) cancers as well as anogenital cancers such as penile, vaginal, vulvar, anal, and cervical cancers. Low-risk HPVs (LR-HPVs) cause cutaneous and anogenital warts (9–12). HR-HPVs include HPV types 59, 58, 56, 52, 51, 45, 39, 35, 33, 31, 18, and 16, which have been classified as carcinogenic, and HPV68 has been classified as carcinogenic, based on epidemiological studies and mechanistic evidence (11, 13).

**Figure 2:**
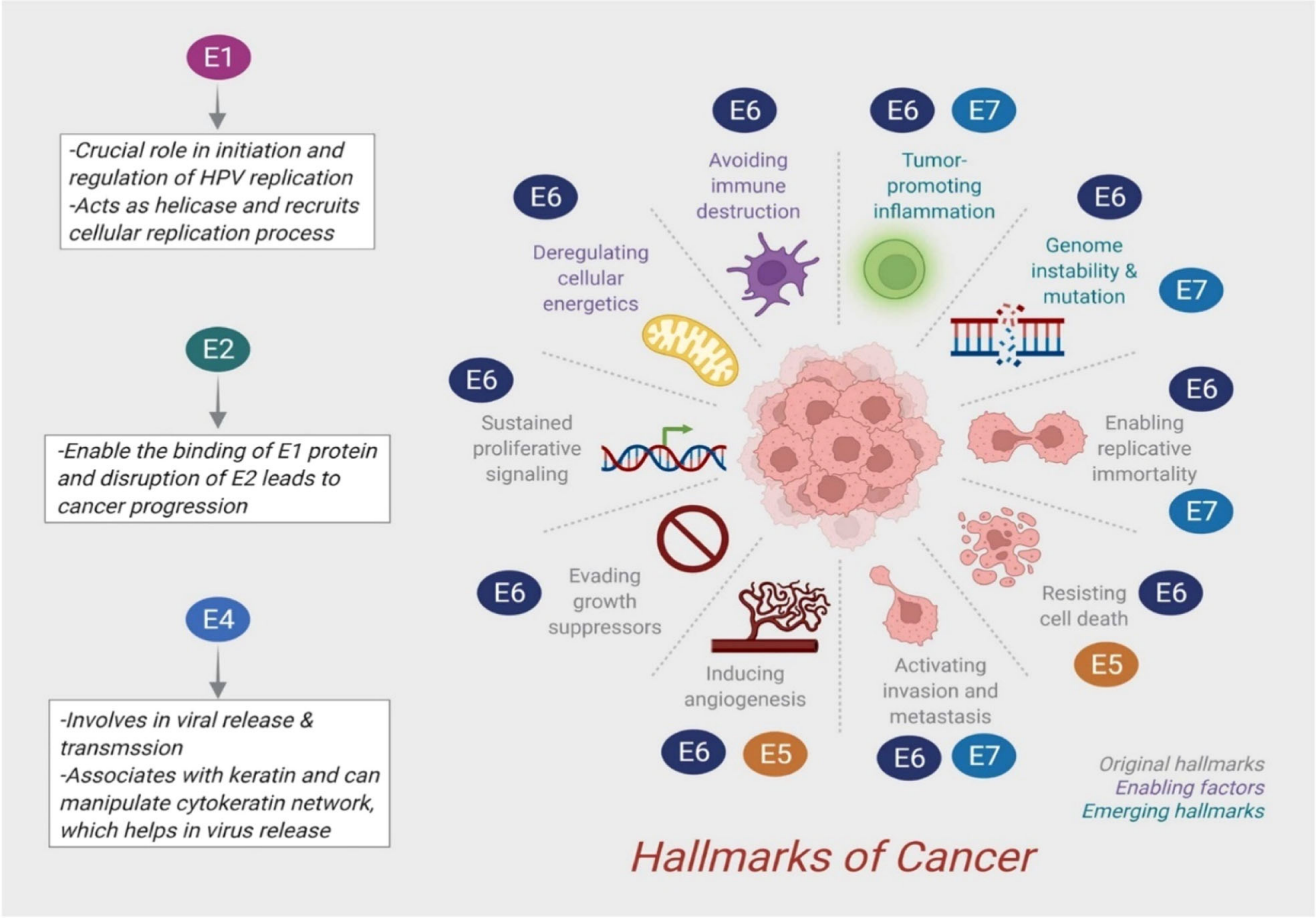
Role of early oncoprotein for cancer progression. Adapted with open access permission from (1).

Cervical cancer caused primarily by HPV infection is one of the largest causes of death in women in southeast Asia (14). HPV16 infection is the key variant responsible for more than 50% of cervical cancer cases in young sexually active women (15). However, 10% of cases of cervical cancer with persistent HPV infection result in cancer progression and invasive carcinoma (16). By monitoring HPV infection through genotyping, women at greater risk of cervical neoplasia can be more accurately identified than by a simple presence/absence test (17). HPV genome constitutes two gene families: early (E) 2 genes encode E1, E2, E3, E4, E5, E6, and E7 proteins, and late (L) 2 genes encode L1 and L2 proteins, which are involved in the progression of carcinoma related to HPV infection (**Figure 2**). **Figure 2** depicts the influence of early proteins over various cancer hallmarks through immune modulation of cellular signaling. Mutations in p53 causes programmed cell death arbitrated by ubiquitin, immunomodulating PIP3K-Akt, Wnt, and EMT pathways through E6 (18). Furthermore, E7 immunomodulation results in the inactivation of pRB, and downregulion of E2F, leading to CC progression (1). Additionally, HPV infections disrupt cytokine production as well as signaling E6 and E7 oncoproteins mediated type I IFN pathway (19, 20). Immuno-response modulators like imidazoquinolones, promotes induction of high levels of type I IFNs activating TLR (toll-like receptor) 7. These high levels of IFNs overcome HPV mediated repudiation of signaling pathway (21). IFN related interactions have been evaluated in the context of the unique W12 cervical carcinogenesis model to determine their relevance in selecting cells with integrated viral DNA in the progression of cancer. In a recent clinical study, episome loss associated with antiviral gene induction is a crucial event in a random selection of cervical keratinocytes carrying HPV16 (22). IFN is administered exogenously to W12 cells containing episomes, leading to the emergence of powerful IFN-resistant cells, the loss of episome-containing cells, and the selection of cells expressing HPV16 with less E2, instead of more E6 and E7 (23). Conclusively, the efficiency of the HPV vaccine to prevent persistent infection is significant although more studies should be needed for long-term effect and it is important to conduct a large-scale cost-benefit analysis to determine whether an approach of this nature is cost-effective (24).

As observed in numerous global areas, the three types of currently licensed HPV vaccinations include nine-valent, trivalent, and bivalent vaccines, which are effective in lowering HPV infection and HPV-related illness incidence (8). Gardasil 9 (Merck Inc.) is a nine-valent (9-V) vaccine that targets HPV18/16/11/6. Gardasil (Merck Inc.) is a four-valent (4-V) vaccine that targets HPV18/16 and also low-risk types HPV11 and 6 that cause genital warts. Cervarix (GlaxoSmithKline) is a two-valent (2-V) vaccine that targets the potentially strong carcinogenic HPV types 18 and 16 (11, 13). The success is explained by the fact that they target and elicit immunity against LR- and HR-HPVs, responsible for 70% and 90% of genital and cutaneous warts and malignancies, respectively (15). Even though HPV vaccines have been demonstrated to be effective, the burden of HPV-related cancer and illness remains significant (8). The investigation of the infections caused by HPV and other accompanying disorders in an epidemiological manner is of utmost importance for measuring and reviewing the three antiviral preventive vaccinations presently available (two-, four- and nine-valent vaccines), and their global adoption (**Figure 2**) (25). Furthermore, at the molecular level, researchers are examining the evolution and characteristics of HPV infections to gain a better knowledge of the true burden of HPV-related illnesses and their repercussions around the world (26). This would enable the discovery of novel treatment technologies for the innovation of next-generation anti-viral vaccines, to overcome the drawbacks of the present preventive regimen, including high prices, limited antiviral protective spectrum, and vaccination management (27–29).

In the current review, we have drawn a comparison between the currently available vaccines, Cervarix and Gardasil, emphasizing the clinical importance of vaccines that are being produced in recent times and shed light on their ongoing clinical trials. Moreover, we have discussed the various therapeutic vaccines and mentioned the challenges faced concerning vaccine coverage, the safety issues of the vaccines, dosage, and the administration routes. Finally, we presented the alternative therapies that are available to tackle HPV infection. This review provides a detailed idea about molecular therapies and cellular landscaping of the vaccines.

## Comparison of cervarix and gardasil

**Figure 3:**
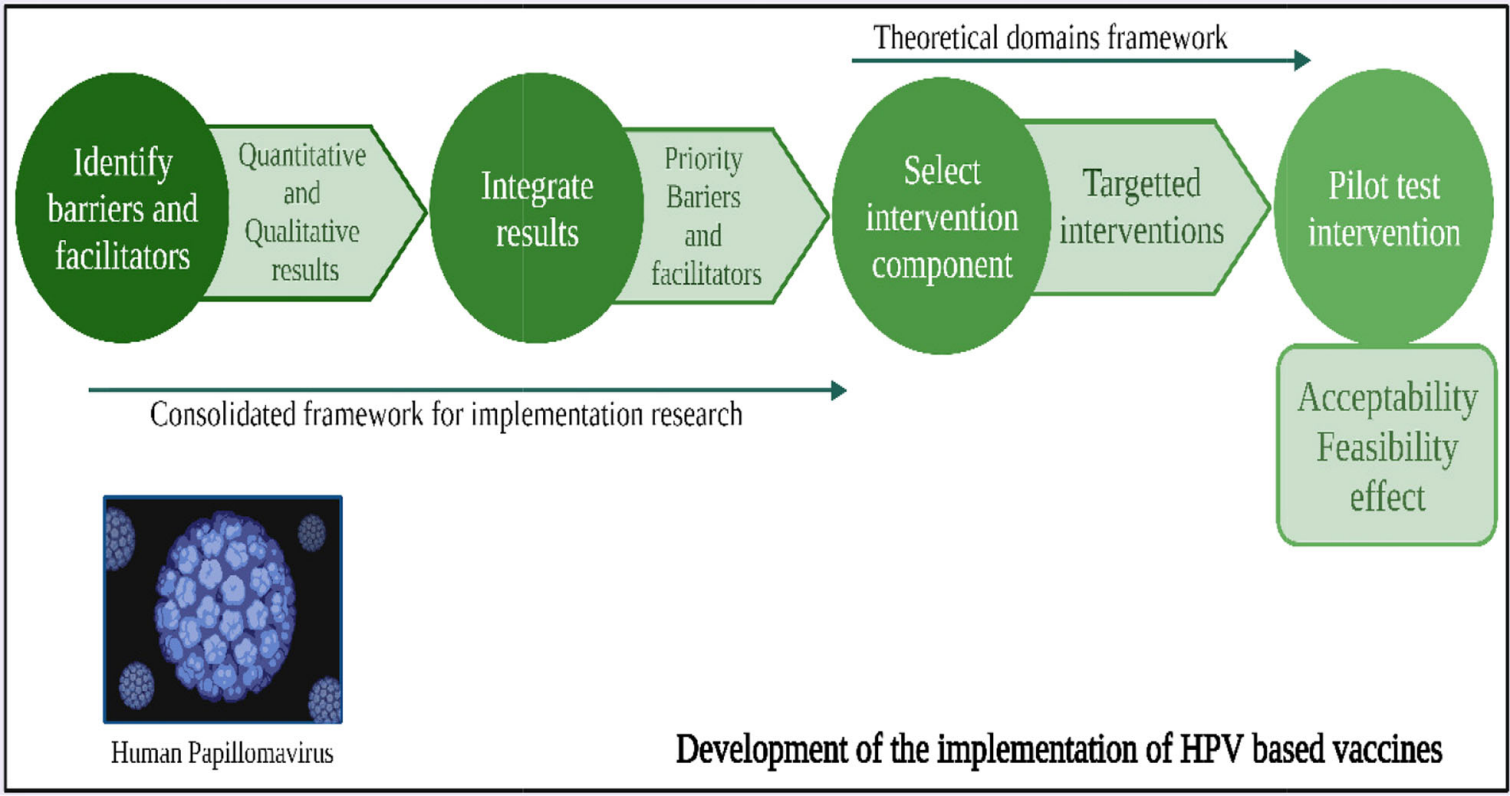
Graphical model showing the development of HPV vaccines over the years to combat related carcinomas.

**Figure 4:**
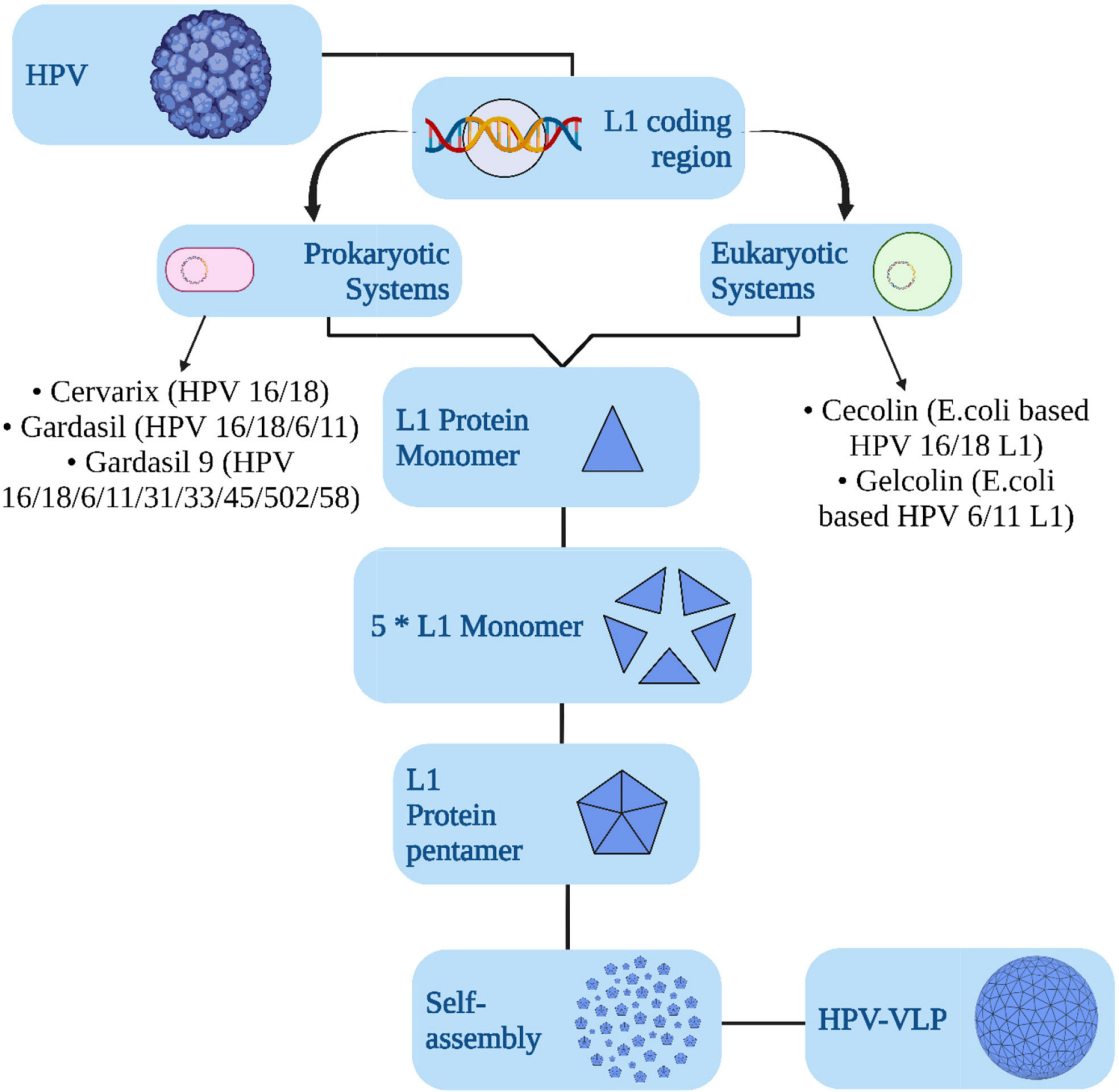
Schematic model of the mechanism of action of a prophylactic vaccine (Cervarix, Gardasil, Ceolin, and Gelcolin) used against HPV infection to combat CC.

The vaccines against HPV are constituted with the aid of recombinant technology using empty protein shells, called virus-like particles (VLPs) of the major capsid protein of papillomavirus, L1 (30) (**Figure 3**). They are devoid of any live organic product or DNA and are deemed to be non-oncogenic and non-infectious, and thus pose less threat than vaccines that are made of attenuated HPV genome (31). The recently developed HPV vaccines are engineered against HPV16 and 18 and are prophylactic vaccines designed for preventive approaches (32, 33). The L1 protein could self-assemble itself into a VLP, which is structurally similar to HPV but without DNA. An immune response generated by these VLPs could produce anti-virion antibodies for protection against future infections (34–36). As observed globally, three types of licensed HPV vaccinations, namely, nine-valent, trivalent, and bivalent vaccines, are effective in lowering HPV infection and HPV-related illness incidence (8). Preventive vaccines are particularly effective in impeding chronic infection and the establishment of neoplasia (**Figure 4**). Prophylactic vaccines aids in decreasing the incidence of HPV related diseases and infections in the future (37). The currently available zero-valent preventive HPV vaccine has been expected to reduce the cases of cervical cancer cases by 90% and of other related malignancies by 50% (laryngeal, oral cavity, oropharyngeal, anal, vaginal, penile, and vulvar) (38, 39). These prophylactic vaccines are first-generation VLP vaccines. Herein, we have elucidated a thorough comparison of the prophylactic vaccines (Gardasil and Cervarix) available to combat HPV infection (40). **Table 1** summarizes the comparison of Gardasil and Cervarix against HPV infection in CC.

**Table 1:** Comparative analysis of Cervarix and Gardasil vaccine against HPV infection.

Features	Cervarix	Gardasil	Gardasil 9	References
Manufacturer	GlaxoSmithKline	Merck & Co.	Merck & Co.	(41, 42)
Valence	Bivalent	Quadrivalent	9 valent	(41, 42)
VLP types	16, 18	6, 11, 16, 18	6, 11, 16, 18, 31, 33, 45, 52, 58	(42–44)
Protection rate against cervical cancer	70%	70%–75%	90%	(42–44)
Adjuvant	MPL absorbed on aluminum hydroxide (AS04)	aluminum hydroxyphosphate sulfate	aluminum hydroxyphosphate sulfate	(45–47)
Expression system	Baculovirus-insect cell	*Saccharomyces cerevisiae*	*Saccharomyces cerevisiae*	(45, 46)
Cross protection	HPV33, 35	HPV31	Unknown	(48, 49)
Sustenance of vaccine efficiency	11 years	10 years	6 years	(50–52)
Adverse effects (AEs)	Localized pain at injection site, inflammation	Localized pain, edema, Muscular pain, dysentery, fever, vomiting	Pain at the localised site, swelling	(53)

Gardasil, manufactured by the American company Merck & Co., is a quadrivalent HPV vaccine and was among the first to be approved by the FDA (41). Gardasil 9, manufactured by the American company Merck & Co., is a nine-valent vaccine licensed by the FDA in 2009 (42). Gardasil offers protection against HPV11 and 6, which cause 90% of genital warts, in addition to HPV18 and 16 (43). Gardasil vaccine is an immunogenic, clinically efficacious, and considerably tolerant in adolescents and preadolescents, according to a clinical follow up study (54). Gardasil has been reported to protect for at least 10 years (50). Gardasil 9 provides protection against HPV58, 53, 45, 33, 31, 18, 16, 11, and 6. This indicates that Gardasil 9 can cover another 20% of CC cases with the extra five HPV types that protect against HPV. Thus, Gardasil 9 can protect against 90% of cervical malignancies (42). Gardasil and Gardasil 9 vaccines are derived from Saccharomyces cerevisiae strain of yeast with aluminum hydroxide as an adjuvant (45). Gardasil protects against HPV infection, genital warts, and precursor lesions of cervical cancer that occur due to the HPV strains covered by it (55–57). Moreover, Gardasil has been shown to reduce HPV infections in the oral cavity, penis, vulva, and anus (58–60). Gardasil possesses a significant hit rate against CIN 2, CIN 2, CIN 3+, and vulvar or vaginal intraepithelial neoplasia of grade II, caused by HPV18 and 16 (61, 62). Other HPV subtypes, however, had a reduced (20%–50%) inhibitory effect on CIN 3+ and CIN 2+ (61, 62). Gardasil had a lower cross-protection impact than Cervarix, with a protective efficacy of 46%, and for HPV58, 52, 45, 33, and 31, the corresponding values were 6%, 18%, 7%, 29% and 46%, respectively (63, 64). Gardasil 9 can effectively prevent precursor lesions and infections caused by multiple types of HPV types at the rate of >95%, injection pre- exposure to HPV (65–67). Gardasil 9 furthermore reduced the incidence of vaginal and vulvar illnesses by 80%–85% and 90%, respectively (10, 66, 68). In a study conducted by Guevara et al. (2018), Gardasil 9 antibodies have been revealed to travel through the placenta, potentially protecting the newborn from HPV11 and 6 infections (69). Gardasil 9 has minute cross-protective efficacy against HPV types not covered by the vaccine and shows a small-scale effect on diseases and infections caused by HPV types other than those covered by the vaccine (67, 70).

Similarly, Cervarix, manufactured in the UK by GSK, is a bivalent HPV vaccination that was licensed by the European Medicines Agency (EMA) in 2007 and the FDA in 2009. Cervarix offers protection against the most frequent HPV oncogenic genotypes (types 18 and 16), which are responsible for roughly 70% of cervical malignancies (44). As an adjuvant, it contains aluminum hydroxide, monophosphoryl lipid A (MPL), and HPV18 and 16 VLPs, collectively known as adjuvant system 04 (ASO4), which is significantly effective against both the HPV16 and HPV18 cervical intraepithelial neoplasia grade 2+ (71). MPL, a toll-like receptor 4 (TLR4) agonist, supports the significant production of antibodies (46). Consequently, another adjuvant, i.e., aluminum hydroxide inorganic component, helps to discharge the intracellular components of the lysed cell and thereby activates the dendritic cells (DCs) to evoke the immune response (72).

Cervarix causes strong anti-HPV18 and 16 antibody titers, which could prevent infection for up to 10 years (48, 64). Cervarix leads to a strong and long-term cross-reactive immune response against HPV45 and 31. In the succeeding three doses of Cervarix, about 85% of individuals developed anti-HPV45 and 31 antibodies, according to a 10-year follow-up research (48). Cervarix (>90%, injection before HPV exposure) offers protection against HPV-related pre-cancerous lesions and abnormalities that are targeted by vaccines, such as adenocarcinoma *in situ* (AIS), cervical intraepithelial neoplasia 3 (CIN 3), and CIN 2 effectively (73, 74). Cervarix is effective (>60%) in the prevention of multiple precancerous lesions, irrespective of previous HPV lesions or infections (62, 75). Moreover, Cervarix exhibits excellent protection against HPV18 and 16 oral infections with 93% reduction in the incidence during a 4-year vaccination intervals (76).

Thus, Gardasil 9 protects the highest number of HPV VLP types (9), followed by Gardasil (4) and lastly Cervarix (2) (**Table 1**). All vaccines provide cross-protection against other VLP types of HPV. Cervarix has the highest sustenance of vaccine efficiency, followed by Gardasil and, lastly, Gardasil 9 (**Table 1**). These are effective in lowering HPV infection and HPV-related illness incidence. However, Cervarix is useful for up to 11 years (51), and Gardasil 9 is effective for at least 6 years against their respective HPV types (52). Reactions on the site of the injection such as edema and pain were the most common adverse effects (AEs) of Gardasil and Cervarix, presumably attributable to inflammation related to the VLP  (53). Diarrhea, myalgia, dizziness, fever, vomiting, and nausea are among the symptoms of Cervarix (53). The most prevalent AEs related to Cervarix are fatigue and headache, which occur in about 50%–60% of all participants (77). Although recipients of Gardasil and Gardasil 9 may experience general symptoms, however zero evidence were reported of any higher risk (53). Since preventive vaccines are particularly effective at avoiding chronic infection and the establishment of neoplasia, to help reduce the incidence of HPV-related diseases and infections in the future. Thus, these three currently available non-valent preventive HPV vaccines have high efficiency in reducing HPV infections, with Gardasil 9 providing the highest protection rate against cervical cancer (**Table 1**).

## Therapeutic vaccine

**Figure 5:**
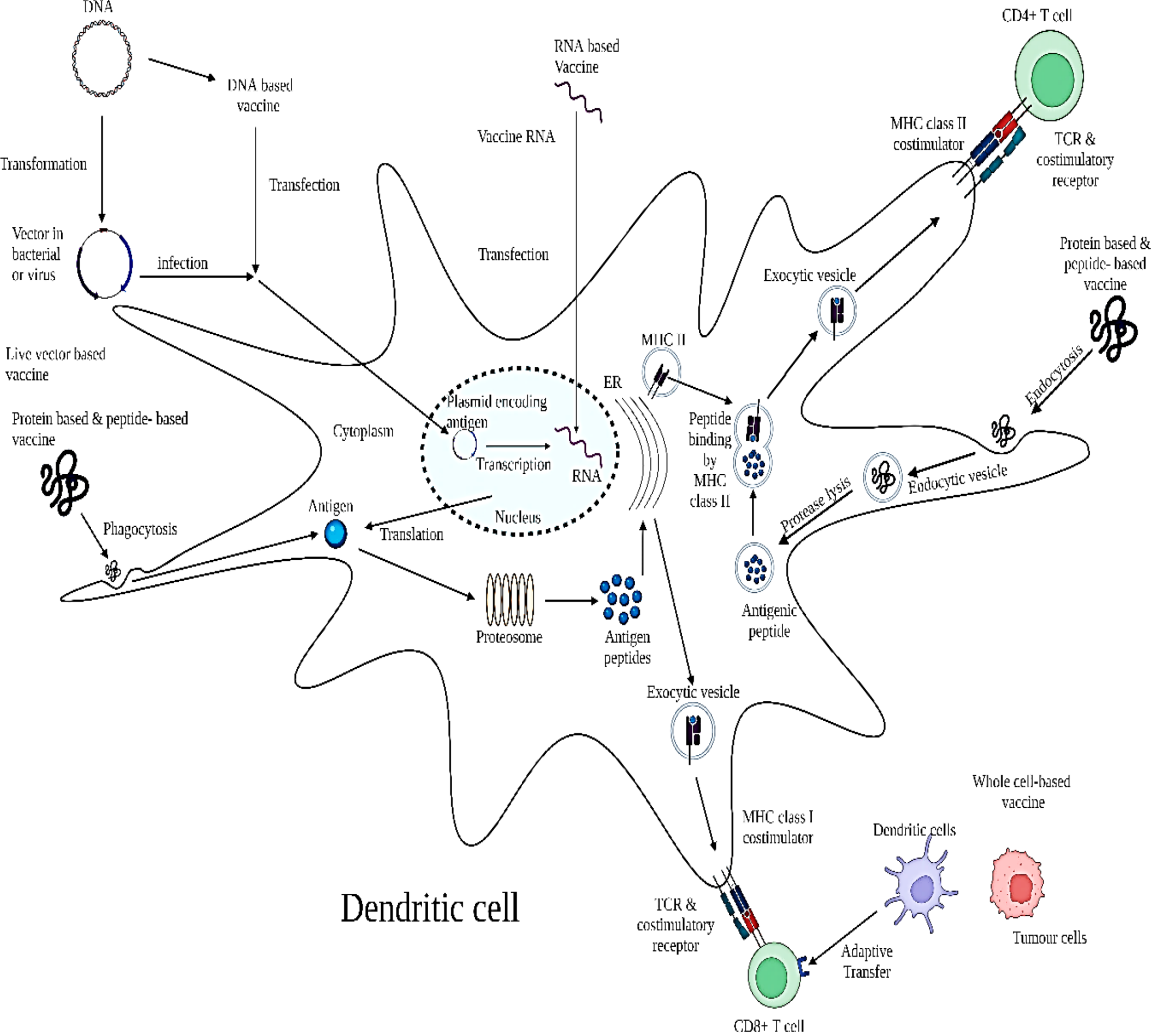
Schematic model depicting the mechanism of action of therapeutic vaccine. The mechanism for anti-HPV activity constitutes through its targeting of E6 and E7 oncoproteins upon APCs for activation of CD8+ and CD4+ cells in the TME. Administration of varying therapeutic HPV vaccine types results in the delivery of different forms of antigen into the body. DNA plasmids encoding HPV oncoproteins E6 and E7 can be transfected into dendritic cells through DNA vaccines or infection of transformed live vector-based vaccines. These antigens are then transcribed into RNA; however, RNA can also be introduced into the cell through RNA vaccines. Transcribed RNA is further translated into antigen proteins or long peptides. Antigen proteins or long peptides can also be taken up by the dendritic cell through phagocytosis after administration of a protein-based or peptide-based vaccine. These proteins or peptides are processed into short peptides by proteasomes and loaded onto an MHC class I molecule in the endoplasmic reticulum (ER) to be presented to T-cell receptors on CD8+ T cells. In addition, dendritic cells or tumor cells can be prepared *ex vivo* to express target antigens on MHC class I molecules with necessary co-stimulatory molecules and be administered back into the body as whole cell-based vaccines through adoptive transfer to prime T cells. On the other hand, the protein or peptide antigens taken up by the dendritic cell can be degraded into smaller fragments by proteases in the endosome. The endosome containing the small antigenic peptides is then fused with the exosome containing the MHC class II molecule, during which the antigenic peptide is loaded onto the MHC class II molecule. The MHC class II–antigenic peptide complex is then transported to the cell surface to be presented to T-cell receptors on CD4+ T cells. Adapted and modified with open access permission from (80).

A wide range of therapeutic vaccines for the treatment of pre-invasive intraepithelial inflammation to severe malignancies has been clinically studied to treat HPV infection (78–81). In clinical studies, prophylactic HPV vaccinations have a significant outcome to prevent HPV infections and associated diseases; however, the number of HPV-associated patients is still high (82). Thus, it is necessary to focus on the significant approaches for the early diagnosis to treat pre- or post-treatment complications of HPV infection (79). At the clinical level, numerous studies have been conducted to examine the therapeutic efficiency of the drug or bioactive molecule to suppress HPV infections. The majority of HPV vaccines are based on targeting viralonco/protein E6 and E7 to act as antigenic receptors on the antigen-presenting cells (APCs) for the activation of antigen-specific CD8+ or CD4+ T cells, respectively (**Figure 5**) (83–85). In antigen-specific T-cell receptor (TCR) activation and immune CD8+ T-cell activation, only the partially digested fragments of E6 and E7 oncoproteins represent the MHC class I molecule of APCs and act as epitopes (80). Herein, we have elucidated the clinical significance of all therapeutic vaccines that could be utilized for HPV infection. **Table 2** summarizes the clinical significance of therapeutic vaccines.

**Table 2:** Clinical significance of different types of vaccines against HPV infection.

Vaccine type	Vaccine name	Phase of trial	HPV infection	Patients	Comments	Side effects	Reference/clinical trial number
Peptide/Protein based	HPV16-SLP	2	HPV16+VIN3	20	Complete response by nine patients, circulation of HPV16 specific T cells among 85% of patients, 83% of patients had CMI against HPV16	Redness, high skin temperature, pain and swelling at vaccine site, fever and chills, tiredness	(86)
		2	HPV16 +HSIL	9	After vaccination, a strong HPV-specific T-cell response was seen in all patients, and changes in the pattern of immune infiltrate	Headache, itching, swelling, redness, reaction at the injection site, fatigue, chills, fever, nausea, diarrhea	(87)
		2	HPV16 + advanced gynecological carcinoma	20	HPV-specific immune response in nine patients	Nausea, fever, chills, flu-like symptoms, injection site reaction, fatigue	(88)
		2	Low-level abnormalities of the cervix	50	HPV16-specific CMI was generated in 97% of patients	Injection site reaction, flu-like symptoms	(89)
			Advanced metastatic or recurrent cervical cancer	18	Scheduled to receive carboplatin/paclitaxel chemotherapy. Proliferative T-cell response was seen in 11 to 12 patients who were vaccinated	Thrombocytopenia, neutropenia, leukopenia, chemotherapy-related anemia, alopecia, gastroenteritis, pulmonary embolism, cancer-related shortness of breath, hydronephrosis, abdominal pain, erysipelas	(90)
	GL-0810	1	Head and neck metastatic squamous cell carcinoma	5	T cell was developed and antibody response was observed among 80% of patients	Itching, erythema, pain at the vaccine site	(91)
	Pepcan + Candin	1	Biopsy confirmed HSIL	31	Histological disease regression was experienced by 45% of subjects	Mild to moderate reaction at the injection site	(92)
	GTL001 (ProCervix)	1	HPV16/18-positive patients having normal cytology	47	Patients in cohort 4 (*n* = 9) experienced a higher rate of HPV16/18 clearance by applying 600 µg of GLT001 powder and imiquimod	Pain, itching, tenderness, swelling at injection site reaction	(93)
	TA-CIN	1	Healthy patients	40	CMI was generated among 25 patients out of 32, TA-CIN-specific IgG in 24 vaccinated patients out of 32	Reaction at the injection site, fatigue, tenderness, headache	(94)
		2	VIN2/3	19	63% lesion response after 1 year of vaccination; in lesion responders, specific CMI was observed	Reaction at injection site associated with imiquimod	(95)
	TA-CIN+TA-HPV	1	HPV16+VIN	10	In two patients partial/complete clinical response was observed		(96)
		2	HPV16 + high-grade AGIN	29	TA-CIN-induced T-cell response was seen in 17 patients, HPV16/18-E6/E7 specific T-cell response was generated in 11 patients, IgG response regarding HPV16-E7 was seen in 14 patients	No side effects	(97)
Nucleotide based	pNGVL4a-sig/E7(detox)/HSP70 + TA-HPV	1	HPV16 + CIN 3	12	HPV16-E7 specific CMI was generated among 58% of patients who were vaccinated, increment of CD8+ T-cell infiltration to lesions	Blister, erythema, pruritus, tenderness, local site reaction	(98)
	pNGVL4a-CRT/E7(detox)	1	HPV16 + CIN 2/3	32	About 30% of patients who were vaccinated experienced histological regression to CIN 1; after vaccination, increment of intraepithelial C8+ T-cell infiltrate	Reaction at the injection site	(99)
	GX-188E	1	HPV16/18 + CIN 3	9	HPV-specific CMI was observed in all patients, by the end of the trial complete lesion regression was demonstrated in seven patients	Swelling, pain at the injection site, hypoesthesia, fatigue, headache, chills, rhinitis	(100)
	VGX-3100	1	HPV16/18 + CIN 2/3	18	Eighteen patients showed HPV-specific CMI, all patients showed HPV-specific humoral immunity	Tenderness, fever, reaction at injection site	(101)
		2b	HPV16/18 + CIN 2/3	167	Regression was demonstrated in 49.5% of patients who were vaccinated as compared to 30.6%, T-cell and humoral responses (102) were enhanced due to vaccinations	Fatigues, myalgia, arthralgia, nausea, erythema, reaction at the injection site	(103)
	DNA (ZYC101)	1	HPV16	12	The immune responses to the peptide epitopes encoded within ZYC101 were raised in 10 of the 12 individuals, and they remained elevated 6 months following the start of therapy.	Back pain, fatigue, influenza-like symptoms, headache	(104)
		1	HPV16	15	Five women showed complete histologic responses, and 11 had T-cell responses specific to the human papillomavirus. Immunoglobulin and anti-E2-specific antibodies were found in four of five full histologic responses.	Back pain, fatigue, influenza-like symptoms, headache	(104)
	DNA (ZYClOla)	2	HPV16/18	127	It was well tolerated by all patients and helped to resolve CIN 2/3 in women under the age of 25.	Reaction at the injection site and pain	(105)
	DNA (pNGVL4a-Sig/E7 (detox)/HSP70)	1	HPV16	15	It is relatedly risk-free and well-tolerated. In patients with established dysplastic lesions, it appears that HPV-specific T-cell responses can be elicited.	Mild transient injection-site discomfort	(105)
	Prime with DNA (pNGVL4a-Sig/E7(detox)/HSP70), boost with recombinant vaccinia virus (TA-HPV) ± imiquimod	1	HPV16/18	75	Study ongoing	–	NCT00788164
Live vector-based vaccines	ADXS11-001	2	HPV16	54	–	–	NCT01266460
	ADXS11-001 administered following chemoradiation as adjuvant treatment	3	HPV16	450	–	–	AIM2CERV
	Live-attenuated *Listeria monocytogenes* vaccine	1	HPV16	15	In end-stage ICC patients, Lm-LLO-E7 infusion was found to be safe and well tolerated.	Chills, vomiting, nausea, pyrexia, headache	(106)

### Peptide- and protein-based vaccines

Different types of viral or non-viral protein or peptide molecules are used as therapeutic agents for immunization (**Table 2**). However, in some clinical studies, the synergistic effect of vaccines with adjuvants is shown to overcome the low antigenicity of protein molecules (79, 107, 108).

The site-specific administration of peptide vaccine induces local effect and suppression of tumor cells activated by either specific or non-specific APC (79), although the selection of a specific T-cell epitope for the peptide vaccines assists the anti-tumor helper T lymphocytes’ (HTLs) and cytotoxic T lymphocytes’ (CTLs) enhanced responses (109). In a mice model, E7 peptide-PADRE peptide and poly (I: C) vaccination have significant E7-specific CD8+ T-cell immunological responses and suppression of TC-1 tumors as compared to the control E7 peptide vaccination. Consequently, the subcutaneous intra-tumoral immunization by the E7 peptide-PADRE peptide and poly (I:C) expresses a higher incidence of E7-specific CD8+ T cells and life span (110). In another study, carrageenan (natural sulfated polysaccharides) has radically expanded the E7 peptide vaccine-specific immune responses *via* the TLR4 activation pathway and improved anticancer effects against E7-expressing malignancies (111, 112).

The clinical trial on the viral protein *Mycobacterium bovis* Hsp65 fused to HPV16 E7 vaccine represented significant efficiency and effectiveness against the high-grade CINs (81). The short peptides after the enzymatic lysis of viral particles act as epitope antigens to stimulate the MHC class I or II pathway and evoke both humoral and cell-mediated immune systems (82). These MHC-represented short antigenic peptides are used to design a vaccine to activate the immune system against HPV (113). Hence, immunotherapy to cause regression of HPV infection was designed through a vaccine utilizing tumor-reactive T-cell peptide epitopes (114). Many adjuvants fused protein vaccines, such as E7-*Bordetella pertussis* CyaA and E7-HBcAg-Hsp65, have been used for *in vivo* immunization and production of HPV-specific CTLs and deterioration of tumor cells (81). Thus, based on the aforementioned studies, it could be concluded that peptide- and protein-based vaccines open up several avenues for future researchers to explore and optimize before they are approved by the FDA for human use.

### Live vector vaccines

The live vectors such as bacterial or viral vectors makes multiple copies of the target antigenic gene or protein in the host depending on the size of delivery molecule (79). The viral-based vaccines are more effective and expressive due to the high propagation rate in the host cell, e.g., adenoviruses, alphaviruses, vaccinia viruses, and fowl pox viruses (115–118).

The vaccinia virus vectors vaccine shows notable HPV infection regression and immunization with using Vaccinia Ankara (MVA) vector that expresses HPV16 E6/E7, CIN 2/3, IL-2, in clinical response (82). At the clinical level phase I–II experiment in progressive cervical cancer patients, a single dose of a live recombinant vaccinia virus expresses HPV16-18 E6/E7 proteins and HPV-specific antibody without any contrary effects (103, 119).

Apart from viral vector vaccines, bacterial vectors are the most developed therapeutic vaccine systems (e.g., *Lactobacillus plantarum, Listeria monocytogenes*, and *Lactococcus lactis*). A clinical phase I study of *Listeria*-based vaccine (Lm-LLO-E7) in 15 cervical cancer patients showed an unadorned effect in 40% of patients (106, 119). In another study, it was reported that *L. monocytogenes* (Lm) is an auspicious live vector that acts as an adjuvant to design and enhance the effect of the vaccine by inducing the macrophages through antigen processing *via* MHC I and MHC II pathways (82). In a phase I/II clinical study, oral administration of GLBL101c drug synthesis from the *L. casei* strain to 27 female patients with CIN 3 resulted in histologic regression in 30% of patients after 9 weeks of treatment. Subsequently, patients who received LEEP (loop electrosurgical excision) displayed a 70% decrease in abrasion to CIN 2. All the patients showed positive outcomes without any adverse side effects of HPV E7 cell-mediated immunity in disease eradication (79, 120). Thus, based on the aforementioned study, it could be inferred that the application of live vectors for vaccine development may provide a new path in the era of the HPV vaccine, even though further clinical studies need to be done (**Table 2**).

### Nucleic acid-based vaccines

A virus consists of the genetic material, the key factor encoding the information for the disease or virulence (121). Therefore, the nucleic acid DNA- or RNA-based vaccines are focused to elicit both cell-mediated and humoral immunity. The long-term therapeutic effect of the nucleic acid vaccine can be studied for the induction of prolonged antigen-specific cellular response and to overcome the self-antigens’ immunological tolerance (122). Moreover, to enhance the effect of the vaccine, different types of assimilators like immunomodulators encoding genetically variant antigenic protein/s within the viral vector have been explored along with DNA vectors (119) (**Table 2**).

The DNA vaccine pNGVL4aCRT-E7 is used in several clinical trials for the treatment of women with CIN 2–3 and is being used for the non-randomized open-label trial analysis (120, 123). In a phase I clinical study, 32 HPV16-associated CIN 2/3 patients were vaccinated intramuscularly, intradermally, or directly into the cervical abrasion with pNGVL4a-CRTE7 (detox), and a calreticulin-related plasmid DNA vaccine showed an immunogenic effect in 69% of patients with less severity at the local administrated tissue/organ (99).

In another study, the HPV DNA vaccine (GX-188E) that targeted HPV antigens through DCs was used, resulting in E6/E7-specific induced IFN-g-producing T-cell response in cervical intraepithelial neoplasia 3 (CIN 3) patients. After 36 weeks of therapy, the proliferation of polyfunctional HPV-specific CD8 T cells, enhancement of cytolytic activity, and the synthesis of effector molecules without any severe side effect at any dose was observed (100). 

Another class of nucleic acid vaccines is RNA-based vaccines containing naked RNA replicons synthesized from alphaviruses to stimulate an antigen-specific immune response (119). The RNA replicon has the ability of self-replication, prolonged antigen expression, and elevated immune response (124). Throughout the transformation of the RNA replicon vector, insignificant chromosomal abbreviations occur in the recipient cell; henceforth, to overcome this drawback of RNA replicon, an integrated DNA vaccine termed “suicidal DNA” is designed to enhance the chromosomal integration (125). The “Suicidal DNA” shows cell apoptosis after the uptake of injected DNA to prevent them from further genetic transformation (126). The commercial mRNA-based vaccine CureVac (Tübingen, Germany) RNActive^®^ has been clinically tested for non-small cell lung and prostate cancer for stimulated long-term humoral and cellular immune responses (119). Furthermore, the Kunjin flavivirus (KUN) vector promotes antigen presentation *via* transected DCs and is secure for patients who have E7-expressing tumors and E7-specific T-cell responses. Conclusively, different RNAs as nucleic acid vaccines are under the clinical trial phase for innumerable HPV and HPV-related cancer development in humans to improve the survival rate (120).

### Whole cell-based vaccines

In therapeutic vaccines, the cell-based vaccines possess the potential outcome to cause regression of HPV-related diseases, by isolating and removing cells (such as T lymphocytes or DCs) from infected donor tissue or pathological sample (127). The biopsy tissue cultured from the tumor and vascular system is modified under *ex vivo* conditions to express immunomodulatory cytokines and is subsequently injected into the host body to cause regression of the infection (120, 128, 129).

HPV16/18-positive advanced cervical cancer patients treated with HPV16 E6 (arm A) or HPV E7 peptide (arm B) showed evoked immune response, with 63% for HPV16 E6 and 58% respectively (**Table 2**). Therefore, pre-immature DCs pulsed and HPV16 E7 or E6 combined effect provide specific immune pathways (119, 130). Furthermore, the strategic outcome of DC vaccines possesses certain limits such as unpredictability in cell differentiation, limited specific donor cell numbers, poor cell transduction activity, and the short lifespan of donor autologous cells (131). In a clinical study, unsuccessful HPV antigen-specific CTL responses were found after the inoculation of pulsed DC-based vaccinations (82).

DC-based whole-cell HPV vaccination is a common and emerging therapeutic vaccination to treat virus-induced cancers (132). DC-based HPV vaccinations containing the HPV antigenic gene or protein acts as a natural adjuvant to induce the antigen-specific immunity and are used in cancer immunotherapy such as siRNA-transfected DCs having pro-apoptotic molecules (133). In a phase I clinical study, DCs carrying HPV16/18 E7 injected into the IL-2 patients resulted in an E7-specific CD8+ response (134, 135). Likewise, DCs with HPV16/18 E7 and Keyhole Limpet Hemocyanin (KLH) trigger the DC maturation in the host body with phase Ib or IIa cervical tumors and increases E7-specific CD8+ T cells (120, 136).

Under *in vivo* conditions, amplified expression of immune modulator proteins was reported in tumor cells succeeding in HPV vaccination. Activation of specific genes for the cytokine IL-2, IL-12, and granulocyte-macrophage colony-stimulating factor (GMCSF) was noticed. However, in clinical trials, these vaccines have been examined to suppress cancer progression, especially for the E6 and E7 malignant tumors. In a clinical study of patients having metastatic melanoma, the instant response of whole cell vaccination along with tumor regression in 50% of patients was reported (82). Thus, these vaccines still might not be successful towards recurring tumors in patients who are positive for HPV with normal cytology or patients with low-grade abrasions.

Based on the aforementioned studies, it could henceforth be concluded that studies on the whole cell-based vaccine open a novel avenue in therapeutic vaccine even though several variables need to be managed in both the clinical and pre-clinical setting before its optimization through FDA.

## Dosage and vaccine administration routes

Another important aspect of the therapy is the dosage and administration of vaccines or drug molecules. Recently, three types of HPV vaccine were clinically used to prevent HPV16- and HPV18-induced genital tract infections (137). The bivalent HPV16/18 vaccine ASO4 is significantly effective against cervical intraepithelial neoplasia grade 2+ with 98% (CI 88–100) efficacy (71). Alternatively, a quadrivalent vaccine against HPV types 18, 16, 11, and 6 (Gardasil^®^, Merck Sharp & Dohme Ltd.) showed a 98% (CI 86–100) efficacy against an HPV16- or HPV18-susceptible population having cervical intraepithelial neoplasia grade 2 or 3 and adenocarcinoma under *in situ* conditions (138). The random clinical testing of HPV adjuvant vaccines was carried out in three-dose regimens over 6 months and established a standard of two doses over 6–12 months. According to the clinical study, WHO recommended the administration of a two-dose schedule to the age group 9–14 years and at least three dose schedules for those aged 16–26 years at least for 6 months to generate antibody responses (137). Besides the WHO standard of three doses for the immunization against HPV infection, research has been conducted to moderate the dosage numbers (139). In 2014, the European Medical Association recommended only two doses of HPV vaccine for adults, followed by the United States after 2-year intervals (140, 141). However, in the clinical trial conducted by the Costa Rica Vaccine Trial (CVT) and the PATRICIA trial, a single dose of HPV vaccine and bivalent HPV vaccine has parallel effectiveness over 4 years in *post-hoc* analysis. Moreover, the insistent immune responses against HPV16 and 18 of CVT are up to five to nine times greater than the natural immunity (32, 139).

Hence, not only the selection of an effective vaccine but also the specific minimum number of doses and administration methodology and period should be focused on for an effective outcome. Essentially, the vaccine can be administered in multiple doses after a regular time interval through injection intramuscularly (IM) or subcutaneously (SC) to evoke the immune system and generation of antigen-specific antibodies (142). Skin is the first line of defense and consists of a huge number of immune cells; e.g., Langerhans cells in the epidermis and DCs in the dermis prevent the pathogen from entering and effectively absorb the antigen to evoke the immune system (143). The intradermal vaccination of peptide- and DNA-based therapeutic HPV vaccines induces serum antibody production by representing the antigenic molecule to macrophages and DCs at minor quantities or doses (144, 145). In a pre-clinical study, a murine model sublingual administration of HPV16 L1 protein vaccine shows significant production of the mucosal secretory IgA and serum IgG comparatively to other delivery methods including intranasal, intravaginal, and transdermal (131). Though the intradermal delivery of therapeutic agents instantly evokes the immune system, certain drawbacks such as pain, post-administration inflammation, edema, and allergic response at the injecting site limit its applicability (146).

## Vaccination coverage and safety challenges

Globally, the market for the HPV vaccine has been growing and numerous commercially available products are clinically used to cause inhibition of carcinoma related to HPV infections (147). In 2006, the first commercialized HPV vaccine approved by US FDA (Food and Drug Administration) was Gardasil (Merck & Co., Kenilworth, NJ, USA), a quadrivalent HPV vaccine that targets HPV6, 11, 16, and 18 infections (148). Next, in 2007, EMA (European Medicines Agency) and the 2009 FDA licensed Cervarix (GSK, Brentford, UK), a bivalent HPV vaccine mostly used to treat HPV oncogenic genotypes HPV16 and 18 to combat cervical malignancies (149). Developed countries have been focusing on reducing design and marketing costs for this vaccine while making tweaks in regulatory matters.

In 2013, an HPV vaccination statistical analysis of the United States showed that 57% of the population aged 13 to 17 years take a single vaccination dose and 38% take triple doses. However, in Australia, 9-year HPV vaccination data from 2007 to 2015 displayed that in the female population of age 15 years, the vaccination rate for single dose was 85.6% and that for the triple dose was 77.4%, which was significantly much higher than that in the United States. Therefore, many non-government agencies are conducting awareness programs, sponsoring medical campaigns, and connecting with society to campaign about HPV vaccinations. Based on this program information, data collection, and outcome, it was reported that HPV illness is mostly found in developing countries, and only 15% of these countries have executed a health program for HPV vaccination (150).

The vaccination trials for 10 years at pre-licensure and post-licensure help monitor and assess confirmation of the commercialized HPV vaccination (151). It was reported that all three HPV vaccination prelicensure studies have positive outcomes without any major side effects (151). The US CDCP (Centers for Disease Control and Prevention) Vaccine Adverse Event Reporting System conducted a large-scale study; i.e., out of 90 million doses of HPV vaccinations, only 7% have AEs. Moreover, no interconnection between HPV vaccinations and other health problems such as ovarian failure, Guillain–Barré syndrome, or postural orthostatic tachycardia syndrome was reported by CDC. Conclusively, HPV vaccinations have tremendous safety scores except for nine HPV vaccines that require regular monitoring (150). Therefore, for HPV vaccine administration parameters, regular upgrading of vaccination in terms of age, dose schedule, and gender has to be performed. Consequently, the research has emphasized the importance of immunizing both genders of any group as well as HPV vaccination from late adolescence to adulthood (152, 153).

## Conclusion and future perspectives

**Figure 6:**
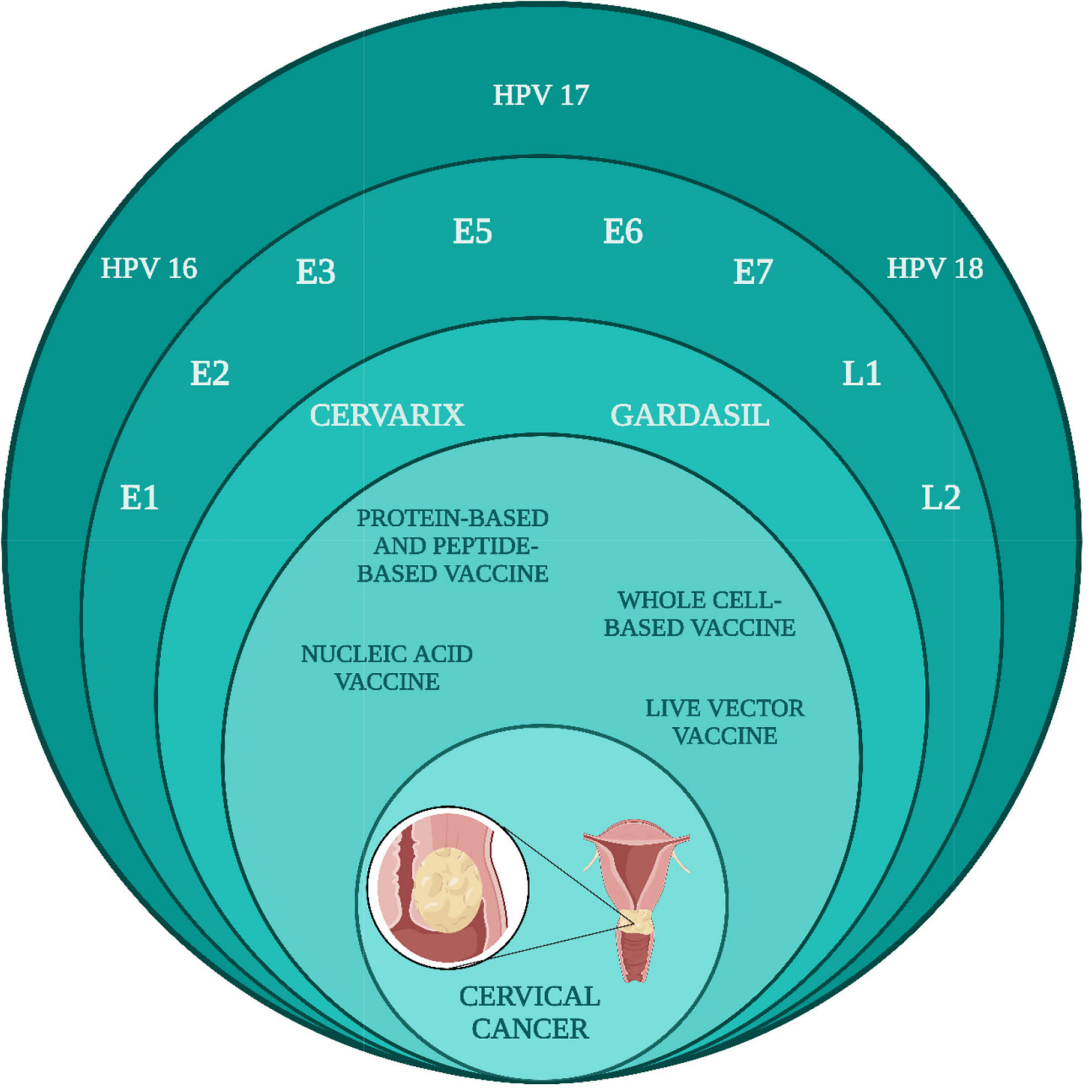
Projection of vaccines constituted from early and late proteins to cause regression against cervical cancer caused due to HPV16, 17, and 18 infections.

HPV is the most frequently occurring STI in the world and is the primary cause of cervical cancer. Early proteins and late proteins are encoded by all types of HPVs and are primarily responsible for CC progression *via* HPV infection [RB1] [LK2]. In the current review, we have covered the currently available vaccines against HPV infection and the vaccines that are under clinical studies against HPV (**Figure 6**). Currently, there are three non-valent prophylactic HPV vaccines, namely, Cervarix, Gardasil, and Gardasil 9. These prophylactic vaccines are first-generation VLP vaccines and have shown efficiency in preventing 90% of cervical cancer cases. Although their clinical trials are very promising, they cannot be termed ideal. These vaccines can be effective if they are given pre-exposure.

The vast majority of therapeutic vaccines target HPV oncoproteins E6 and E7, to deliver E7 and E6 antigens to APCs in various ways to activate HPV antigen-specific CD8+ cytotoxic T cells or CD4+ helper T cells. Peptide/protein-based chemicals account for the vast majority of published evidence on therapeutic vaccinations. Due to their low antigenicity, these vaccines are frequently combined with immunogenic adjuvants in clinical trials and provide the benefits of safety and stability. Combining numerous epitopes in peptide-based vaccines has the potential to improve peptide-MHC binding and specific T cell-mediated protection against HPV-infected cells. Live vector-based vaccines, which use viruses or bacteria as the vector, reproduce within the body, making it easier for the antigen to disseminate.

Clinical trials are ongoing for live vector-based vaccines; however, most are in phase I or II. More research is required on these types of vaccines. DNA-based vaccines are safe, simple to fabricate, and capable of eliciting both CTL and Th as well as B-cell immunity and offer long-term protection. Immunomodulators are used to improve immunogenicity. Numerous RNA vaccines for HPV-related cancers have progressed to clinical trials; nevertheless, more work in the creation of HPV RNA vaccines is necessary. For HPV-related disorders, whole cell-based vaccinations have been developed as a potential therapeutic vaccine. HPV vaccines based on DCs have emerged as possible therapeutic vaccinations against HPV-related cancers. DC-based vaccinations have a variety of drawbacks, including unpredictability in vaccine quality due to changes in cell culture protocols, difficulty in obtaining large quantities of autologous DCs from patients, low DC transduction efficacy, and the short lifespan of autologous DCs. Even though pulsed DCs induced HPV antigen-specific CTL responses, DC-based vaccines failed to yield clinical responses. Tumor cell vaccines have the disadvantage of producing new tumors in patients, limiting their therapeutic application, especially in HPV-positive patients with normal cytology or patients with low-grade abrasions.

The coverage and dosage administration of the vaccines have also been covered in the current review. Patients should receive two doses of the HPV vaccination over 6–12 months, according to current recommendations. Following two doses of HPV vaccines provided at least 6 months apart to adolescents aged 9–14 years, WHO recommends a two-dose schedule for 15-year-old girls. Three doses were non-inferior to two doses in women aged 16–26 years in whom the vaccine’s efficacy was demonstrated. When delivered *via* the intradermal method, DNA- and peptide-based therapeutic HPV vaccines have also been shown to produce a favorable immune response. Although intradermal administration is associated with reduced discomfort at the time of administration, post-delivery local effects such as redness and duration due to inflammation at the injection site are more common.

Conclusively, cost-associated studies fortify the vitality of economic analyses in determining resource allocation, especially in public health, and further support evidence-based decision-making when considering public health interventions and other prevention programs. Immunization against HPV is considered a cost-effective cervical cancer prevention implementation. The non-valent vaccine produces greater health benefits than the bivalent and quadrivalent vaccines at a lower societal cost. Furthermore, considering herd immunity, any considerable expansion in coverage will aid in declining cancer incidence and healthcare costs. The health benefits of vaccines have been proven to be a clever investment from multiple perspectives. As novel vaccines hit the market targeting morbidity, quantification of disease burden and modeling of the cost-effectiveness of intervention options turn out to be more important. Current models are successful in predicting cost-effectiveness; however, there is a necessity for revisions in clinical evaluations for the quadrivalent and bivalent HPV vaccine. If properly implemented, there can be a successful reduction of the HPV burden globally.

## Author contributions

The manuscript was conceptualized and written by RB. ADh, AD, and LK collected the information and analyzed it. All authors participated in editing, writing, and proofreading. TM worked on the illustrations. The manuscript was finally cured, modified, and supervised by RG and SM for the submission. All authors contributed to the article and approved the submitted version.

## Funding

This research was supported by Basic Science Research Program through the National Research Foundation of Korea (NRF) funded by the Ministry of Education (NRF-2020R1I1A2066868), the National Research Foundation of Korea (NRF) grant funded by the Korea government (MSIT) (No. 2020R1A5A2019413), a grant of the Korea Health Technology R&D Project through the Korea Health Industry Development Institute (KHIDI), funded by the Ministry of Health & Welfare, Republic of Korea (grant number: HF20C0116), and a grant of the Korea Health Technology R&D Project through the Korea Health Industry Development Institute (KHIDI), funded by the Ministry of Health & Welfare, Republic of Korea (grant number: HF20C0038).

## Acknowledgments

The authors acknowledge the respective departments and institutions for providing facilities and support. The authors would also like to appreciate Biorender.com for their software.

## Conflict of interest

The authors declare that the research was conducted in the absence of any commercial or financial relationships that could be construed as a potential conflict of interest.

## Publisher’s note

All claims expressed in this article are solely those of the authors and do not necessarily represent those of their affiliated organizations, or those of the publisher, the editors and the reviewers. Any product that may be evaluated in this article, or claim that may be made by its manufacturer, is not guaranteed or endorsed by the publisher.

## Abbreviations

HPV, human papilloma virus; STI, sexually transmitted infection; GLOBOCAN, Global Cancer Observatory; WHO, World Health Organization; E protein, early protein; L protein, late protein; HR-HPV, high-risk HPVs; LR-HPV, low-risk HPVs; VLP, virus-like particles; FDA, United States Food and Drug Administration; EMA, European Medicines Agency; MPL, monophosphoryl lipid A; TLR4, toll-like receptor 4; AIS, adenocarcinoma *in situ*; CIN 3, cervical intraepithelial neoplasia 3; VIN/VaIN 2+, vulvar/vaginal intraepithelial neoplasia grade 2; APC, antigen-presenting cells; TCR, T-cell receptor; MHC, major histocompatibility complex; CTL, cytotoxic T lymphocytes; HTLs, anti-tumor T lymphocytes; MVA, Modified Vaccinia Ankara; Lm-LLO-E7, *Listeria*-based vaccine; ELISPOT, enzyme-linked immunospot; ASO4, 3-O-desacyl-4'-monophosphoryl lipid A; CVT, Costa Rica Vaccine Trial; IM, intramuscularly; SC, subcutaneously; CDC, US Centers for Disease Control and Prevention; ATC, adoptive T-cell therapy; HPB242, MRP 2,4-bis (p-hydroxyphenyl)-2-butenal; pRB, protein retinoblastoma.
